# *Bacteroides fragilis* Lipopolysaccharide and Inflammatory Signaling in Alzheimer’s Disease

**DOI:** 10.3389/fmicb.2016.01544

**Published:** 2016-09-26

**Authors:** Walter J. Lukiw

**Affiliations:** Bollinger Professor of Alzheimer’s disease (AD), Neuroscience Center and Departments of Neurology and Ophthalmology, Louisiana State University Health Sciences Center, New Orleans, LAUSA

**Keywords:** 42 amino acid amyloid-beta (Aβ42) peptides, Alzheimer’s disease, bacteroidetes, DAMPs and PAMPs, lipopolysaccharides, microbiome, NF-kB, systemic inflammation

## Abstract

The human microbiome consists of ~3.8 × 10^13^ symbiotic microorganisms that form a highly complex and dynamic ecosystem: the gastrointestinal (GI) tract constitutes the largest repository of the human microbiome by far, and its impact on human neurological health and disease is becoming increasingly appreciated. Bacteroidetes, the largest phylum of Gram-negative bacteria in the GI tract microbiome, while generally beneficial to the host when confined to the GI tract, have potential to secrete a remarkably complex array of pro-inflammatory neurotoxins that include surface lipopolysaccharides (LPSs) and toxic proteolytic peptides. The deleterious effects of these bacterial exudates appear to become more important as GI tract and blood-brain barriers alter or increase their permeability with aging and disease. For example, presence of the unique LPSs of the abundant Bacteroidetes species *Bacteroides fragilis (BF-LPS)* in the serum represents a major contributing factor to systemic inflammation. BF-LPS is further recognized by TLR2, TLR4, and/or CD14 microglial cell receptors as are the pro-inflammatory 42 amino acid amyloid-beta (Aβ42) peptides that characterize Alzheimer’s disease (AD) brain. Here we provide the first evidence that BF-LPS exposure to human primary brain cells is an exceptionally potent inducer of the pro-inflammatory transcription factor NF-kB (p50/p65) complex, a known trigger in the expression of pathogenic pathways involved in inflammatory neurodegeneration. This *‘Perspectives communication’* will in addition highlight work from recent studies that advance novel and emerging concepts on the potential contribution of microbiome-generated factors, such as BF-LPS, in driving pro-inflammatory degenerative neuropathology in the AD brain.

## The Human Microbiome and *Bacteroides fragilis*

*Homo sapiens* and their complex microbiome, consisting chiefly of bacteria with microbial eukaryotes, archaea, fungi, protozoa, viruses, and other microorganisms making up the balance, together compromise the entire metaorganism whose symbiotic associations and host interactions are critical to human health and disease (Hugon et al., 2015; Seksik and Landman, 2015; Youssef et al., 2015). There are approximately 52 recognized divisions of bacteria, however, humans, have co-evolved with – 2 dominant phyla: Bacteroidetes [about 20% of all gastrointestinal (GI) tract bacteria] and Firmicutes (about 80%); with Actinobacteria (about 3%), Proteobacteria (~1%), and Verrucomicrobia (~0.1%) making up significantly smaller fractions. These four major bacterial divisions represent the ‘bacterial-core’ of the human microbiome (Hakansson and Molin, 2011; Zhao et al., 2015; Hug et al., 2016; Sender et al., 2016). About 98% of GI tract microbiota consists of anaerobic bacteria, and Bacteroidetes species, constituting ~30% of all GI tract bacteria are the most abundant Gram-negative bacteria found in the GI tract outnumbering *Escherichia coli* abundance by at least 100 to 1 (Fathi and Wu, 2016; Foster et al., 2016; Hug et al., 2016; Rogers and Aronoff, 2016; Sampson and Mazmanian, 2016; Sender et al., 2016).

Bacteroidetes species such as *Bacteroides fragilis*, normal commensals of the GI tract, are thought to be generally beneficial to human health via their production of polysaccharides, volatile fatty acids, cleavage of dietary fibers into digestible short-chain fatty acids, and other nutrients, however, when they escape this environment they can cause substantial inflammatory pathology with significant morbidity and mortality (Hofer, 2014; Khanna and Tosh, 2014; Fathi and Wu, 2016). Dietary intake may have a role in regulating the composition and stoichiometry of the GI tract microbiome; for example Bacteroidetes species have been observed to proliferate in porcine models fed high-fat diets deprived of sufficient dietary fiber (Heinritz et al., 2016). In addition to their lipopolysaccharide (LPS) generation, *B. fragilis* endotoxins are a leading cause of anaerobic bacteremia and sepsis/systemic inflammatory distress through their generation of the highly pro-inflammatory zinc metalloprotease metalloproteinase *B. fragilis* toxin (BFT) *fragilysin* (Choi et al., 2016; Fathi and Wu, 2016). BFT has recently been shown to disrupt epithelial cells of GI tract barriers via cleavage of the synaptic adhesion zonula adherens protein E-cadherin (Seong et al., 2015; Choi et al., 2016; Zhan and Davies, 2016). It is currently not well understood if GI tract barrier-disrupting proteolytic endotoxins such as BFT are able to propagate pathogenic actions via the systemic circulation to further disrupt the blood-brain barrier and transfer LPS, BFT, and other endotoxins into the cerebrovascular circulation to the neural cells and synaptic circuitry of the CNS. *B. fragilis* has been shown to play a pathological role in neurodevelopment including autism spectrum disorder (ASD) via circulating metabolites (Hofer, 2014). It has also recently been reported that along with BFTs amyloid peptide-dependent changes in synaptic adhesion affect both the function and integrity of synapses, suggesting that the observed deficits in synaptic adhesion in Alzheimer’s disease (AD) play key roles in the progressive disruption of functional signaling throughout neuronal networks (Lin et al., 2014; Seong et al., 2015; Leshchyns’ka and Sytnyk, 2016).

## Inflammatory Signaling in Alzheimer’s Disease (AD)

Multiple and highly interactive aspects of increased inflammatory signaling is a consistent and recurrent feature of AD and the major pathological lesions that define AD, including insoluble Aβ42-enriched peptide deposits, neurofibrillary tangles, apoptotic, damaged, and dying neurons, and activated microglia are potent neuropathological stimulants that maintain the brain in a chronic and self-reinforcing inflammatory state (Hill and Lukiw, 2015; Calsolaro and Edison, 2016; Minter et al., 2016; Richards et al., 2016; Varatharaj and Galea, 2016). These progressive and ultimately fatal pro-inflammatory and neurodegenerative processes appear to be further stimulated by aberrant or excessive deregulation of the innate-immune response, and an increasing focus has been placed on pathological contributions by the human microbiome including dietary effects on microbial composition that appear to support pathological functions (Hill and Lukiw, 2015; Zhao et al., 2015; Foster et al., 2016). There is a wealth of accumulating evidence (a) that specific types of microbial LPS and endotoxins (such as BF-LPS and BFT) from enterotoxigenic microbes specifically impact microglial-mediated innate-immune responses, phagocytic, and detoxifying mechanisms and amyloidogenesis characteristic of inflammatory neurodegeneration (Bhattacharjee and Lukiw, 2013; Hill et al., 2014; Clark and Vissel, 2015; Hill and Lukiw, 2015; Lim et al., 2015); and (b) a resurgence of our interest in the importance of GI tract and blood-brain barrier systems that normally exclude these microbiome-sourced toxins from the systemic circulation and CNS but become leaky with age and disease (Montagne et al., 2015; Choi et al., 2016; Minter et al., 2016; Richards et al., 2016; Soenen et al., 2016; van de Haar et al., 2016; Varatharaj and Galea, 2016; Zhan and Davies, 2016; Zhao et al., 2016).

Interestingly, while secreted LPS, proteolytic endotoxins, and amyloid monomers are generally soluble as monomers over time they form into highly insoluble fibrous protein aggregates that are implicated in the progressive degenerative neuropathology of several common, age-related disorders of the human systemic circulation and CNS including systemic inflammation response syndrome, multiple sclerosis (MS), prion disease, and AD (Asti and Gioglio, 2014; Clark and Vissel, 2015; Devier et al., 2015; Richards et al., 2016; Zhao et al., 2016). At the genetic level, virtually all of this inducible inflammatory signaling within the CNS involves NF-kB activation and NF-kB-recognition and binding to target NF-kB DNA sequences as a prelude to the up-regulation of pro-inflammatory gene expression pathways, including the up-regulation of discrete families of pro-inflammatory pathogenic microRNAs that selectively down-regulate their mRNA targets (Lukiw and Bazan, 1998; Lukiw et al., 2008; Devier et al., 2015; Zhao et al., 2016).

## Lipopolysaccharide (LPS) and LPS Signaling

Lipopolysaccharides are characteristic components of the outer leaflet of the outer membrane of Gram-negative bacteria shed into the extracellular space that play key roles in host–pathogen interactions of the innate-immune system (Hill and Lukiw, 2015; Zhao et al., 2015; Jiang et al., 2016; Maldonado et al., 2016). While LPSs contain large and hypervariable polysaccharide/oligosaccharide regions, the relatively conserved lipid region (lipid A) is the endotoxic and biologically active moiety that is responsible for septic shock (Jiang et al., 2016; Maldonado et al., 2016). A “canonical” LPS structure is represented by that of LPS from *E. coli*, that contains one of the most potent neurotoxic lipid A species known, consisting of a 1,4′-biphosphorylated glucosamine disaccharide bearing six fatty acids which are unbranched chains 12–14 methyl(ene) units in length. Other ‘*lipid A*’ species show variability in the number, length, and composition of the attached fatty acids, as well as variability in the degree of phosphorylation and number and types of substituted phosphate ligands. For instance, *BF-LPS lipid A* is penta-acylated and mono-phosphorylated and contains branched fatty acids 15–17 methyl(ene) units in length; deviations from the canonical lipid A structure are known to have a profound impact on the host innate-immune responses. LPS activates Toll-like receptors (TLRs), membrane-spanning protein receptors expressed in microglial cells of the innate immune system which recognize common damage- or pathogen-associated molecular-patterns (DAMPS, PAMPs; Gustot et al., 2013; Land, 2015; Maldonado et al., 2016; Minter et al., 2016; Richards et al., 2016; Varatharaj and Galea, 2016). TLRs play key roles in host protection from microbial invasion via the activation of the innate-immune system by sensing structurally conserved DAMPS or PAMPs from microbes or microbial exudates that are distinguishable from, and not innate to, the host organism (Land, 2015; Yu and Ye, 2015). Interestingly, of the 13 currently characterized TLRs the microglial TLR2 and TLR4 are activated by amyloid, LPS, lipoglycans and/or other microbial triggers that subsequently induce cytokine production, inflammation, phagocytosis and innate immune defense responses that directly impact the development of CNS pathology. More specifically, the TLR2 complex can recognize biofilm-associated LPS and amyloids produced by both Bacteroidetes and Firmicutes (Nishimori et al., 2012; Bhattacharjee and Lukiw, 2013; Asti and Gioglio, 2014; Hill and Lukiw, 2015). Interestingly, Aβ42 peptides overproduced in AD that are associated with microglia-mediated inflammatory responses have very recently been shown to activate TLR2 and/or TLR4 (Hill and Lukiw, 2015; Yu and Ye, 2015; Minter et al., 2016). Of further interest is: (a) that microbial amyloids induce pro-inflammatory interleukin IL-17A and IL-22, triggers for NF-kB activation/signaling and cyclooxygenase 2 activation via direct TLR2 activation (Nishimori et al., 2012); and (b) that increased levels of both IL-17A and IL-22 are associated with age-related inflammatory neurodegenerative diseases such as AD (Zhang et al., 2013; Calsolaro and Edison, 2016; Maldonado et al., 2016; Richards et al., 2016). Bacterial endotoxin- and LPS-induced neuro-inflammation has been known for some time to be important in driving the generation of Aβ42 (Lee et al., 2008; Asti and Gioglio, 2014; Hill and Lukiw, 2015; Zhao et al., 2015). In addition to TLR2 and TLR4 at least one additional microglial transmembrane LPS receptor CD14 mediates phagocytosis of both bacterial components and Aβ42 peptides, hence expanding roles for microglia and microglial LPS receptors in the pathophysiology of AD (Lee et al., 2008; Halmer et al., 2015; Jiang et al., 2016). Of further interest is that gram negative bacterial exudates such as BF-LPS are hypervariable in composition, and different Bacteroidetes species appear to generate unique temporal patterns of LPS that exhibit rapid adaptive changes – these include the modulation of LPS synthesis and structure and alterations in DAMP/PAMP recognition features as strategies for host immune system evasion (Land, 2015; Maldonado et al., 2016; Richards et al., 2016).

## Concluding Remarks

**FIGURE 1:**
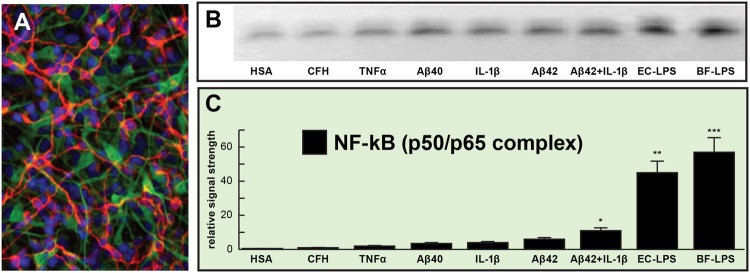
**Relative induction of NF-kB (p50/p65)-DNA binding in pro-inflammatory factor and lipopolysaccharides (LPS)-treated primary human neuronal-glial (HNG) co-cultures. (A)** HNG cells in primary co-culture for 1.5 weeks; HNG cells were stained with a neuron-specific β-tubulin III (red fluorescence λ max~650 nm; anti-βTUBIII antibody, Sigma-Aldrich, St Louis, MO, USA); an antibody against glial fibrillary acidic protein (GFAP; green fluorescence; λ max ~510 nm; Santa Cruz Biotechnology, Santa Cruz, CA, USA) and DAPI nuclear stain (blue fluorescence; λ max~470 nm; Thermo Fisher Scientific, Waltham, MA, USA); 20×; **(B)** induction of the pro-inflammatory transcription factor NF-kB (p50/p65 activation complex) by various physiologically relevant, pro-inflammatory factors all at equal dosage (25 nM); NF-kB abundance was measured by NF-kB-DNA binding assay (a measure of NF-kB activation and binding to NF-kB-DNA recognition sequences) onto a 36 nucleotide end-labeled double stranded DNA fragment containing the canonical human NF-kB (p50/p65) recognition sequence 5′-GGGGACTTTCCC-3′ as previously described (Lukiw and Bazan, 1998; Lukiw et al., 2008; Devier et al., 2015; Clement et al., 2016); a scrambled control nucleotide containing no such NF-kB recognition sequence showed NF-kB-DNA binding activity (data not shown); **(C)** data from gel bands in panel **(B)** quantified in bar graph format**;** note robust induction of the NF-kB (p50/p65 complex) by *Escherichia coli* lipopolysaccharide (EC-LPS; LPS from *Escherichia coli* 0111:B4; Sigma L3012, St Louis, MO, USA) or *B. fragilis* lipopolysaccharide (BF-LPS; prepared by methods previously published; Eidhin and Mouton, 1993) that was ~45- to ~55-fold higher than that of the control human serum albumen (HSA) protein, and was fivefold to sevenfold higher than the combination of the pro-inflammatory Aβ42 peptide and IL-1β together (at 25 nM each); complex mixtures of microbiome bacterial LPS on NF-kB induction might be expected to be additive or synergistic; HSA = (control) human serum albumen; CFH = complement factor H; TNFα = tumor necrosis factor alpha (cachectin); IL-1β = interleukin 1-beta; Aβ40, Aβ42 = amyloid beta peptide, 40 and 42 amino acids in length; EC-LPS, BF-LPS = *E. coli*, *Bacteroides fragilis* lipopolysaccharide; error bars represent one standard error of the mean; *N* = 4; *p < 0.05; **p < 0.01; ***p < 0.001, ANOVA.

We hope that this ‘*Perspectives*’ article has adequately highlighted some recent findings on microbial-derived LPS and proteolytic endotoxins and has engendered interest in the potential contribution of these neurotoxic and pro-inflammatory microbial exudates to amyloidogenesis and age-related inflammatory neurodegeneration. Taken together, these current observations advance seven key areas in our understanding of the role of the microbiome in progressive, age-related inflammatory neurodegeneration: (a) that relatively low, nanomolar amounts of bacterial LPS are extremely potent inducers of the pro-inflammatory transcription factor NF-kB (p50/p65 complex) in human primary human neuronal-glial (HNG) co-cultures of brain cells (**Figure 1**); (b) that different LPS preparations from different bacterial species appear to exhibit slightly different trends in the induction of an inflammatory response, as quantified by the extent (mean values) of NF-kB activation and DNA-binding (**Figure 1**); (c) that the proliferation of microbial species such as the BF-LPS and BFT generating Bacteroidetes may be regulated by diet, environment, and lifestyle factors such as dietary fiber intake can impact neurological health, CNS inflammation, and degenerative disease (Hofer, 2014; Khanna and Tosh, 2014; Zhan and Davies, 2016); (d) that LPS transit across compromised GI tract and blood-brain barriers underscore the critical roles of cellular adhesion structures in allowing passage of noxious molecules from the GI tract into the systemic circulation and CNS (Montagne et al., 2015; Soenen et al., 2016; van de Haar et al., 2016); (e) that biophysical, gastrointestinal, and neurobiological barriers which may become more ‘leaky’ with aging again underscore the important role of tight junctions in moderating systemic and CNS inflammation and immune-mediated inflammatory disease (Hill and Lukiw, 2015; Varatharaj and Galea, 2016); (f) that DAMPS and/or PAMPs common to both LPS, endotoxins, and/or amyloid may trigger TRL2, TRL4 and/or CD14 microglial receptors to propagate and sustain inducible AD-relevant inflammatory responses within the CNS (Land, 2015; Zhao and Lukiw, 2015; Varatharaj and Galea, 2016); and (g) that LPS abundance, speciation and complexity in the CSF and/or blood serum may be useful diagnostically for the onset of mild cognitive impairment (MCI), the clinical precursor for the development of AD. Clearly, more research into the intriguing realm of human microbiome-host interaction is warranted, and the study of the complex interactions between each biological niche is certain to shed new light on the still evolving concepts and mechanisms of *microbiome interplay and control* in human neurological health and disease.

## Author Contributions

WL and the late Dr. James Hill performed experiments, researched and wrote this paper; the author is sincerely grateful to colleagues and collaborators for helpful discussions, medical artwork and unpublished data; the contributions of these researchers have been recognized in the ‘Acknowledgements’ section.

## Conflict of Interest Statement

The author declares that the research was conducted in the absence of any commercial or financial relationships that could be construed as a potential conflict of interest.
